# Implementation of sugar-sweetened beverages tax and its perception among public health stakeholders. A study from Poland

**DOI:** 10.3389/fnut.2022.957256

**Published:** 2022-07-29

**Authors:** Katarzyna Brukało, Krzysztof Kaczmarek, Oskar Kowalski, Piotr Romaniuk

**Affiliations:** ^1^Department of Health Policy, Chair of Public Health Policy, School of Health Sciences in Bytom, Medical University of Silesia, Katowice, Poland; ^2^Department of Human Nutrition, Chair of Dietetics, School of Health Sciences in Bytom, Medical University of Silesia, Katowice, Poland

**Keywords:** sugar sweetened beverages, sugar tax, stakeholder involvement, Poland, SSB tax, food policy

## Abstract

**Background:**

One of tools to tackle growing problem of overweight and obesity are the taxation mechanisms applied to sugar-sweetened beverages, which are expected to influence the common eating behaviors, but also they have impact on the market and public finances. The solution is therefore highly entangled in the complex of social and intersectoral interests generating a number of opportunities and threats affecting its feasibility.

**Aims:**

The study aims to depict the views of Polish stakeholders on the implementation of the sugar tax in Poland, particularly the perception of success determinants, barriers, as well as views on the features of the implemented solutions and possible alternatives.

**Methods:**

We used semi-structured interviews with 18 individuals representing key public health stakeholders in Poland. The interview consisted of four parts, where first concentrated on the advantages and disadvantages of the SSB tax, the second part explored stakeholder involvement and stances, third concerned the feasibility of the project, and in the fourth part respondents were asked for suggestions for decision-makers regarding the content of the project and its implementation process. To reconstruct position of 4 main political parties we applied desk research. We used MAXQDA v2020 to analyse the collected data.

**Results:**

Stakeholders tend to expressed conflicting views on the effectiveness, relevance and socio-economic impact of the SSB tax. All of them agreed that the tax may appear severe for the poorest groups, children and adolescents, while disagreeing about the economic impact of the levy. The allocation of additional tax revenues was raising doubts, with stakeholders believing that the fiscal aim is the basic reason for implementing the tax, while these resources should be primarily dedicated to health promotion intervention and prevention of diet-related diseases. On the other hand, the political debate on the tax was highly superficial with strong populism arising of the presented positions.

**Conclusions:**

There is a need to conduct a thorough public debate and improvements in terms of public communication to increase social awareness, sealing and refining the implemented solutions. Close cooperation with market players and non-governmental organizations is highly recommended.

## Introduction

The problem of excessive body mass is an urgent and growing challenge for modern public health worldwide. Despite numerous interventions, its impact on public health systems has increased in recent decades. Since 1975 the incidence of obesity has almost tripled (1). In the European Union in 2019, 52.7% of the population suffered from overweight or obesity. In Poland, the epidemiological studies suggest the status to be even worse than the EU average, with overweight occurring in 52.4% of men and 32% of women, and with 16.5% of men and 16.2% of women suffering from obesity (2). Due to existing data, in 2019 12% of Poles consumed sugar-sweetened beverages on a daily basis, which is the fourth highest result in the European Union, and additional 43% consumed this kind of soft drinks at least once a week (3). The consumption is especially high in case of younger age groups and males, but with no significant differences between income groups.

Overweight and obesity is a multidimensional issue. Excessive body mass is correlated with several health problems, including a higher risk of cardiovascular diseases, type 2 diabetes, hypertension, or some types of cancer (1). Besides its direct impact on health, obesity constitutes a major challenge for social and economic policies. In the first of these two fields, the basic burden relates to obese peoples' increased risk of social exclusion with all its consequences, including a negative bilateral correlation between the occurrence of obesity and economic status and level of education, as it has been confirmed in the studies in developed countries (4).

Economic policies appear as a battlefield, with various solutions being applied to tackle the issue of obesity among citizens, including a sort of financial instrument intended to discourage consumers from buying certain products. These may appear as the differentiation of the value added tax (VAT) rate, excise duty on selected products, or the implementation of additional charges on products with excessive sugar content (the so-called sugar tax) or saturated fat content (the so-called fat tax). These taxation solutions are intended to affect consumption (limiting the consumption of taxed products) and consequently improve health status (5).

Sugar-sweetened beverages (SSB) have been identified as a product whose consumption significantly contributes to the prevalence of overweight and obesity while being highly caloric with negligible nutritional value for the body (6–8). The volume of consumption of SSB varies significantly between countries, appearing visibly higher in Western European countries (9). The problem is also clearly visible in Poland, where in 2019, as many as 12% of residents over 15 years of age consumed sugar-sweetened drinks every day, which was the fourth highest result in the EU (10). As the frequency of SSB consumption tends to decrease with age, policies limiting their intake are especially crucial when addressing children and adolescents to limit the risk of overweight and obesity among these age groups (11).

The undeniable advantage of taxing SSBs is the fact that it is considered an effective tool to reduce consumption, being at the same time relatively highly feasible (11, 12). For both health and economic reasons, such solutions were implemented in many European countries, including Belgium, Finland, France, Ireland, Latvia, Norway, Portugal, the United Kingdom, Hungary, and the region of Catalonia (Spain).

In Poland, a decision to introduce a sugar levy covering SSBs was also taken by the Government, with the first conceptual proposal being presented in 2018. In 2019 it was officially announced as a part of the National Cancer Strategy (13). The new tax was supposed to be implemented on 1 July 2020; however, it was postponed to 1 January 2021 due to the Covid-19 pandemics outbreak (14). The tax structure assumes an additional fee for beverages with added sugars (monosaccharides, disaccharides, foodstuffs containing these substances) or sweeteners (e.g., xylitol, sorbitol or aspartame, i.e., “light” and “zero” drinks), as well as to drinks with the addition of taurine and caffeine (i.e., energy drinks). Although it is obvious that the burden of this fee falls on consumers, it is intended to be paid by beverage producers per the following adopted algorithm:

- PLN 0.50 (0.11 EUR)^1^ fixed fee—for the beverages with sugar content equal to or less than 5 g per 100 ml of drink or for the content of at least one sweetener in any amount.
- PLN 0.05 (0.011 EUR) (see text footnote 1) variable fee—for each 1 gram of sugars above the 5 g per 100 ml of drink threshold.

A lower fee is charged for beverages with sugar content above 5 g per 100 ml, if they contain fruit, vegetable or fruit and vegetable juice of at least 20% of the raw material composition, or if they are carbohydrate-electrolyte solutions (e.g., isotonic drinks) (14).

An additional fee of PLN 0.10 (0.022 EUR) (see text footnote 1) per 1 liter is charged on drinks with the addition of taurine or caffeine (e.g., energy drinks). However, the maximum amount of the sugar fee may not exceed PLN 1.20 (0,26 EUR) (see text footnote 1) per 1 liter of drink.

The construction of the Polish tax is similar to the one implemented in Portugal and United Kingdom (15, 16). However, a specific feature of the Polish solution is that it covers also the sugar free soft drinks and those with taurine and/or caffeine added. The official justification for this solution is to prevent the raising consumption of energetic drinks among adolescents and to inhibit the trend of solidifying bad nutritional habits. The Government while submitting the new tax proposal to the Parliament, justified charging the sugar-free drinks with the tendency to increase the tolerance of sweet taste in consumers, regardless of the sweetening substance used, as well as with the fact of higher appetite due to the consumption of sweetened drinks, which indirectly cause the increased energy supply, regardless of the caloric value of the drink itself (17).

Noticeably, the introduction of the tax is not accompanied by additional solutions related to package labeling that could increase consumer awareness about the added sugar content in the product. The only existing requirements are that the information about the total amount of sugar per 100 g/100 ml of the product is being given, as is required by the EU law (18).

The SSB tax law provides additional revenues to be distributed between the National Health Fund and local governments, which are expected to utilize part of these funds for health education and health promotion, including interventions related to the prevention of overweight and obesity (14).

Implementing the sugar tax is a political process which brings certain consequences. Its implementation produces the best results if its rationale and aims are understandable, acceptable, and preferably supported by all the stakeholders, including first and foremost, consumers themselves (19). Since both the possibilities of implementing the sugar tax and stakeholders' views depend on the economic and political situation, as well as the socio-cultural context, learning from international experiences and the exchange of insights appears to be valuable potential determinants of the policy success (20, 21). Furthermore, recognizing and understanding the stakeholders' positions and views is crucial in identifying barriers and facilitators accompanying the tax implementation (22, 23), which constitutes the basic rationale for the present study.

This study aimed to present the views of Polish stakeholders representing a wide spectrum of sectors (health care, industry, politics, science, etc.) regarding the implementation of the sugar tax in Poland, including the perception of factors determining its success, possible barriers, as well as the views on features of the implemented solutions and possible alternatives. This research was conducted as a case study constituting part of the Work Package 6 of the Policy Evaluation Network (PEN) project (24).

## Material and methods

The study was conducted between July 2020 and July 2021. To identify and recruit key stakeholders from all relevant sectors (academia, political scene, economy, health care, consumer organizations, etc.), we used a combination of purposive sampling and snowball sampling. In addition, the interviewees were asked to indicate individuals whose statements should also be considered to ensure the complete picture of the SSB tax implementation in Poland. The sample size was determined following the principle of saturation, i.e., the recruitment of subsequent stakeholders was carried out until the data was collected, allowing for an in-depth understanding of the issues discussed, and no new information appeared in the interviews (25). A total of 33 stakeholders were invited to participate in the study, of which 18 agreed to be interviewed. Detailed information is presented in Table 1.

**Table 1:** Recruitment of stakeholders.

**Sector**	**Stakeholders approached (*****n** =* **33)**	**Stakeholders declined** **(*****n** =* **15)**	**Stakeholders included** **(*****n** =* **18)**
Ministries and public institutions	7	No response (*n =* 2) No time (*n =* 1) Insufficient knowledge of SSB taxation (*n =* 2)	2 (Ministry of Health and National Institute of Public Health)
Professional associations	2	NA	2 (Polish Public Health Society and Polish Society of Dietetics)
Retailers and industry associations	5	No response (*n =* 1) No time (*n =* 1)	3 (Association of Sugar Producers in Poland, Association of Polish Regional Breweries, Polish Federation of Food Producers)
Academia	5	No response (*n =* 2)	3 (Academics in the field of psychodietetics, economics and management in health care, preventive dentistry)
NGO and patients/consumer associations	7	No time (*n =* 4)	3 (Polish Association of Diabetics, Consumer Federation, NGO in the field of nutrition and health)
Health professional associations	2	NA	2 (Main Chamber of Nurses and Midwives and Supreme Medical Council)
Politics experts/consultants	5	No response (*n =* 1) No time (*n =* 1)	3 (former Ministry of Health, Provincial consultant in the field of diabetology, economic expert)

We applied the semi-structured interview method, having constructed the scenario of the interview based on a literature review conducted before the study, concentrating on the studies exploring stakeholder opinions regarding the tax on sweetened beverages in other countries (23, 26, 27). The interviews were conducted by K.B. and K.K. They were divided into four main parts. The first part concentrated on the advantages and disadvantages of the SSB tax in the Polish context and possible means of reducing disadvantages and maximizing advantages. The second part explores the issue of stakeholder involvement, especially those that may foster or block the pro-healthy taxation-related initiatives. The third part concerned the feasibility of the taxation project, including existing barriers and suggested ways to overcome them, as well as opportunities and favorable circumstances that may leverage the feasibility. In the final part, respondents were asked for suggestions for decision-makers regarding the content of the project and its implementation process. The interview included a separate set of questions specific to the declared stance against the project. The interviews were conducted in the form of recorded conversations, with the participants' informed consent and acceptance. Each interview lasted between 40 and 90 min. Prior to the interview the stakeholders were given a background material about the construction of SSB tax implemented in Poland, along with the information on how the revenues are planned to be spent.

Each interview was recorded, and transcripts were sent to the interviewee concerned to guarantee accuracy (28). The analysis of the collected material was performed using the MAXQDA software v. 2020. Interview transcripts were anonymised and analyzed according to a thematic approach (28). Two independent researchers (K.B. and K.K.) carried out the analysis to establish the code tree. The verification of the correctness of the data encoding and resolving differences in coding and interpretation were done by P.R and O.K. The initial set of codes is included as Supplementary Material 1.

The largest political parties in Poland were also invited to participate in the study (8). A total of 27 invitations were sent, including 15 to party secretariats and their regional branches and 12 to individual politicians. Since none of the parties or their representatives agreed to take part in the study, we decided to reconstruct their positions based on desk research, with a special focus on the meeting of the Parliamentary Health Committee on 13 February 2021 (29), where the discussion on the proposed SSB tax project took place. Therefore, we adopted the meeting transcripts as material reflecting the views and positions of the individual parties or coalitions represented in the Polish Parliament (*N* = 4). Although the transcript accurately reflects the parties' positions against the sugar tax, it does not fully follow the questions included in our interview scenario. For this reason, we decided to analyse this part separately, which is reflected by the structure of the “Results” section of the paper below.

## Results

### Impact of the SSB tax on SSB purchases and consumption, health-related outcomes, and products' reformulation

According to interviewed stakeholders, implementing the tax will affect the supply and consumption of SSB beverages, but only to a limited extent, with only the Association of Sugar Producers presenting a different view. The Ministry of Health stressed that “*price is the third factor, along with quality and taste, determining the choice of food products*.” Therefore, introducing an additional fee may effectively prevent diet-related diseases. However, it should be emphasized that most academic and policy experts and industry and scientific organizations point to the risk of a substitution effect: “*Beverages are a common good. Therefore, as the price of beverages rises, consumption will decline. The possible substitution effect can be expected in its natural form - there will be a reduction in consumption of beverages which will become more expensive, in favor of consumption of beverages which will be relatively cheaper*.”

In the opinion of the respondents, a real reduction in overweight and obesity in Poland can also be expected as a result of the reduced consumption; however, this will appear only in the long term, although, as emphasized by the producers' associations, “*the substitution phenomenon that is emerging does not appear as the change from the wrong (sweetened beverages) to the right (spring/mineral water) eating habits. Consumers will continue to consume sweetened beverages within their price range (and perhaps of much lower quality)*.” Moreover, as scientific societies emphasize, obesity and overweight are caused not only by an excessive supply of simple carbohydrates but also by numerous other factors, including an excessive supply of fats, physical inactivity, and poor behavioral patterns. Therefore, the SSB tax as such, “*yes—it will likely reduce the incidence of overweight and obesity, but the overall BMI cannot be expected to be significantly reduced*.“ On the other hand, the Ministry of Health appeared to be more optimistic, assuming that ”*the levy on sweetened beverages is likely to improve body mass index across all groups, with the greatest impact being felt in those of the lowest socioeconomic status*.”

All respondents agreed that the implemented SSB tax would be particularly severe for the poorest groups, as well as for children and adolescents because they do not have any income. This, in a way, should be considered a desirable effect because, as mentioned by Polish Association of Diabetics: “*according to experience, poorer groups are more likely to make nutritional mistakes and inherit bad habits*”. Scientific associations even emphasize that making it more difficult for any group to access sweetened beverages should not be seen as a disadvantage but as a positive aspect of the SSB tax because: “*it is impossible to imagine any individuals who should supplement sugar for legitimate reasons. We know of diseases that require its restriction, but no disease requires its supply*.”

Economists and representatives of public bodies point to two more groups that will be particularly affected by the implementation of the SSB tax, namely food service operators and producers of sweetened beverages; however, they all agree that in the end, the entire cost will be passed on to the consumer.

Perhaps this is why most respondents do not expect spectacular reformulations of recipes either. A political expert points out that “*there is a ‘cutting straw' competition among manufacturers of sweetened beverages, so where the lowest price could be achieved has already been done. The sugar content in drinks of well-known brands has already been significantly reduced. So, these large producers are not only ready to take such steps but have even done so themselves by adapting to consumer expectations and market demands*.” Therefore, it can be concluded that the highest price increase will be on the cheapest products.

### Economic consequences of the SSB tax on the health sector and the SSB industry

As economists emphasize, the implementation of the SSB tax is not strong enough to affect the national economy. The effects, if any, will be observed on a macro scale—in the area of a particular market. Some producer associations emphasize that the impact of the tax on GDP will not be significant, as “*decreases in sales of artificially sweetened beverages will be compensated by purchases of other beverages, such as natural fruit juices*.” Economists also expect similar shifts within the so-called supply chain, where a switch can be expected from the sugar producers' market to the sweeteners' market. On the other hand, according to estimates from producers' organizations, between 20,000 and 40,000 people in the sweetened beverage industry would lose their jobs (adding to the crisis caused by the COVID-19 pandemic), mainly representatives of small and medium-sized enterprises. Another threat, according to producers, is the distortion of market competition: “*Polish entrepreneurs will be obliged to pay such a tax, while abroad such solutions do not function everywhere*.” There is a strong polarization in opinions. On the one hand, some economists emphasize that additional fiscal burdens are economically ineffective. On the other hand, experts from politics, academia and patient organizations believe that “*economic consequences are irrelevant. The most important thing is that the end justifies the means, and the goal is to reduce disease burden*.”

Despite the described disagreement and inconsequence in declared views in some cases, there is a consensus regarding the allocation of additional revenue generated through the new tax. All stakeholders agree that these resources should be dedicated to health promotion intervention and prevention of diet-related diseases, but mainly to educational activities. There are also suggestions that the funds should also be dedicated to the monitoring of the health impact of the tax, including consumer surveys and research on changes in food composition and changes in consumption of given food categories and sugar intake (ministries/institutions) and to initiate discussion on the impact of carbohydrate-rich products, especially sucrose, on oral health and overall body condition (academic expert).

Policy experts and health professional associations point out that additional funding will support a system that has been underfunded for many years. However, producer associations emphasize that, in reality, the additional financial burden is only an indirect tool to feed the national budget, as “*it can be expected that a certain part of the money from the budget will not go to the National Health Fund because it will be fed by the stream of money from the sugar tax*.”

### Public perception of the sugar tax

Respondents from all sectors were unanimous concerning this aspect, stating that the sugar tax would arouse public resistance. More specifically, two main reasons were pointed out: lack of education and low public awareness and general public reluctance to pay more new taxes. However, academic experts and representatives of the health care sector emphasized that they will likely weaken over time, as “*public opinion notices such problems only on an ad hoc basis, i.e., when the tax is being implemented and when there is a lively discussion. I am also not convinced that it impacts consumer choices*.”

Industry representatives tend to cite surveys they have conducted, in which up to 80% of respondents were against the implementation of the SSB tax. “*In the course of the consultations carried out, arguments were raised by consumers who are aware and leading a healthy lifestyle, why, although they use such drinks only occasionally, they should contribute to the cost of treatment of people who repeatedly overuse them. At this point, one can come to an absurd conclusion that the tax should be paid on obesity*.” An argument has also been raised that the introduction of the SSB tax is interference with personal freedom or freedom of choice. And in this regard, experts agree that “society is a kind of consensus” and “*if we treat this issue from the orthodox liberal perspective, then any incentive and any tax can be treated as an interference with freedom”* and the analogous “*restriction of access to alcohol, cigarettes or sweetened beverages is fully justified from the point of view of state policy*.”

The industry organizations have argued that a mandate-based policy approach is not as effective as a health education approach. All experts identified the need to raise consumer awareness to build public acceptance of the steps taken. According to the respondents, the consumers play a crucial role because they, with their individual consumer choices, are responsible in the first line for overweight and obesity. In the case of children and adolescents, these are also the parents. The lack of health education was mentioned as the responsibility of “*all those involved in health care in Poland, but the responsibility for monitoring the scale of the problem and effective prevention is assigned to the leaders*,” hence the main expectation addressed toward, e.g., the Ministry of Health, or Ministry of Education. However, the SSB tax can be a helpful instrument because, as academic experts emphasize, although “*the main influence is exerted by incorrect individual decisions, there is a lack of appropriate legislative solutions to support consumers*.”

### Sugar tax—stakeholders' positions: Reducing opposition

Many organizations and institutions are expected to be involved in implementing the SSB tax. Governmental institutions such as the Ministry of Health (which should be the initiator of the tax), but also the Ministry of Finance, Education or Sport, as well as the National Health Fund, which is the main payer for health services in the system, have been mentioned as the main stakeholders expected to support the implementation of the tax. In addition, positive lobbying is also expected from the Patient Ombudsman, patients' organizations, educational institutions (such as kindergartens and schools), and all social organizations.

As emphasized by economists, implementing the sugar tax is “*a classic game of interests. On the one hand, we have the state apparatus represented by institutions, offices, and ministries, and on the other hand, we have producers who can organize themselves and exert more or less influence on decisions*.”

However, the role of food producers, who, for obvious reasons, may show resistance to additional financial burden on their products, is not so clear-cut. Study participants indicate that “paradoxically, many producers of sweetened beverages may be hidden allies of this tax. Such supporters are especially found among manufacturers who are already prepared for its implementation” and among “*organizations of the socially responsible manufacturers.”* Nevertheless, natural resistance should be expected to occur in the industry. Healthcare representatives and patient organizations expressed that it would encourage manufacturers if tax relief is proposed “*to enable them to bring their products into compliance with the law”* or “*tax reliefs for maintaining employment or switching to healthy components*.” Meanwhile, the producers, in addition to financial relief as incentives for risk-taking and product reformulation, indicate that a substantive conversation based on the exchange of concrete arguments is needed to break down resistance. This kind of strategy is also favored by the Ministry of Health, which emphasizes that “*it is important to undertake reliable information activities even before the commencement of legislative work, as well as to organize wide public consultations with the industry affected by the regulation. It may also be helpful to prepare a strategy for countering industry objections*.” The Ministry of Health also expects aggressive marketing actions taken by sweetened beverage producers, which may influence public perception and strengthen social resistance; therefore, “*it is important at the stage of conceptual work on a new fee to create a professional literature review and undertake public information activities at the level of preliminary legislative work*.” And in this respect, the role of the media in initiating and creating public debate may prove to be decisive. However, at the same time the expert and industry community expect “*reliable and smog-free information*” from the media and “*comments without political overtones, because health is a superior good, common to all, regardless of views*.”

### Barriers related to tax implementation

Apart from the obvious conflict of interests, the respondents pointed to the lack of social dialogue, which should involve three fundamental parties: legislators, consumers, and producers. There is even a deeper problem, as “*there is a weakness of our system to sell certain solutions positively, while there is much evidence that it works better to “stimulate” the citizens and treat them as partners than to prohibit*.” On the other hand, according to economists, “*eventual public consultations are just an illusion - they have no real impact on the amount of the tax or the subject of taxation*” “*the state has the advantage over every citizen that it can forcefully impose certain solutions, and the tax is exactly this kind of specific abstraction*.” Also, the experience of patient organizations shows that often the implemented changes are not a response to real needs, where “*for example, the health care and system changes are often made based on ‘magical' tables, instead of consultation with patients*.”

The Ministry of Health also admitted that the main barriers are the failure to reach an agreement with manufacturers related to the lack of a transition period and the lack of agreement on the fee amount.

In the opinion of the manufacturers, a sine qua non condition for an agreement and elimination of barriers is not only a broad social dialogue including all stakeholders but also an in-depth assessment of the effects of the regulation and its principles, namely the subject of the taxation, as “*one may have the impression that the catalog of products covered by the additional tax was not part of a well-thought-out and planned strategy*”, as well as the way and the amount of the levy.

### Opportunities and favorable circumstances

Experts agree that the undeniable opportunity in favor of implementing the tax was the position of the government and parliamentary majority, which guaranteed support for this legislative act. The Ministry of Health also emphasizes that not only the political consensus was decisive, but also the support of experts, adequate communication and the reasonable allocation of the revenues generated with the tax are undeniable circumstances favoring implementing the tax. Patient organizations point out that these actions align with “*a kind of 'fashion for healthy nutrition' observable among some population groups and can be a good starting point for educating society*.” The expert community stresses that a reliable analysis of the dietary patterns in terms of the sources of sugar consumption, consumer research concentrating on the factors determining consumer choices, as well as the cooperation of experts, political and industry environments would help develop a commonly acceptable consensus.

### Suggestions for changes and overall perceptions of the construction of the SSB tax

According to experts, the timing of the tax implementation and its fast-track legislative procedure are subject to concern. Health care industry organizations emphasized that the state of the epidemic resulting in the implementation of far-reaching social and economic restrictions was not a good moment to impose additional taxation fees. Manufacturers, on the other hand, indicated that at least a 2-year transition period would be necessary, although, as patient organizations responded, “*we have been discussing the tax for several months, so manufacturers knew the tax was coming into effect and they had enough time to prepare*.”

Another issue requiring consideration is the subject of taxation. On the one hand, the inclusion of beverages with non-sugar sweeteners added as a subject of taxation was supported by patients' organizations because “*up to now there is no common official position on the harmfulness (or not) of the use of sweeteners. Therefore, it is good that they have been included, first, because of their potential harm; secondly, because of the consolidation of unhealthy habits*.” The manufacturers' organizations also supported this regulation: “*We assume that sugar as a natural product is better for humans than chemical sweeteners*.” On the other hand, healthcare organizations and economists were much more liberal in their positions: “*In my opinion, this tax should be implemented in small steps. Hence, I do not get the idea of taxing zero beverages and those sweetened with non-sugar. If we prefer such aggressive measures, let us put taxes on calories, which is unrealistic*.”

Respondents take a highly diverse stance on the amount of the additional fee. Producer associations argue that the fee is too high, while other stakeholders stress that “*there are three characteristics of an effective tax. Firstly, it must be precisely defined; secondly, it must be sufficiently severe and precisely levied; thirdly, it must be difficult to circumvent*.” Therefore, they suggest that the tax rate should be as high as 20–25% and should depend on the sugar content in beverages.

Manufacturers would expect opposite policy solutions. Instead of implementing a sugar tax, a good solution would be to reduce VAT on mineral and spring waters or use the money to subsidize fruits and vegetables. This proposal is consistent with the opinion of economists, who are in a position “*that this tax is just a fiscal measure, which can be very conveniently justified with the concern about the health of the citizen*,” but the same goal can be achieved through the regulation addressing food composition.

### The value of international recommendations and implementation in other countries

Respondents justify their statements by referring to the experience of other countries; however, they tend to do so in a highly selective way. Supporters of the SSB tax are limited to just a general statement that the experience of other countries shows that this tool is effective, while the opponents claim that there is existing evidence that this solution is ineffective.

The representatives of the industry organizations are raising specific examples. They point out that, according to the PricewaterhouseCoopers [PWC] Report, wherever the SSB tax was implemented, a substitution effect appeared, where people continued to consume sweet drinks but started to buy those that were cheaper. In England and Mexico, a short-lived effect of reduction in the SSB consumption was followed by a reverse effect, namely an increase in consumption. Thus, in countries where the sugar tax was implemented and where the regulation on composition requirements was not considered, sugar consumption did not decrease. Producers were also doubtful about the tax rate: “*according to our analyses, the Polish consumer was to pay three times more than the French consumer in terms of consumer purchasing power*.”

Industry representatives suggested learning from international experience. For example, the Croatian model, where drinks sweetened with little sugar and sweeteners are not additionally taxed, or completely changing the approach in line with the German reform, under which a whole system of incentives for a healthy lifestyle was implemented, including 0% VAT on wellness and fitness services, a 500-euro tax-deductible amount if spent on services for a healthy lifestyle.

### SSB as a subject of political discussion and the political parties' positions

Four coalition parties from the Polish Parliament actively participated in the political debate: Prawo i Sprawiedliwość (*The Law and Justice*; PiS), Lewica (*The Left*), Konfedracja (*The Confederation*), and Koalicja Obywatelska (*The Civic Coalition*; KO).

Four main issues dominated the political discussion before implementing the SSB tax, namely, the advantages and disadvantages of the solution, its potential effects and the procedure of implementing the tax.

The ruling party only emphasized the advantages of the new tax [Law and Justice, PIS], which initiated the policy. First of all, the support of experts in the field was highlighted, including the declaration of full support from the World Health Organization. Similar solutions successfully implemented in Europe were also raised as an argument. Additionally, it was emphasized that the Polish representatives of the medical community spoke with one voice in favor of the tax, despite political divisions and sentiments.

The exclusion from taxation of medical devices, registered dietary supplements, special-purpose food, infant formulae, and excise goods was also raised as an advantage, which was supposed to prove that the implemented solution had been carefully considered and designed.

An often-emphasized strength of the policy was allocating potential revenues obtained from the sugar tax. According to the project's assumptions, half of the amount should go to the National Health Fund for strictly defined preventive and educational purposes and the other half to local governments, with the requirement to be used for the same purpose, namely health education and prevention, especially concentrating on excessive body weight in children and adolescents and its possible health consequences.

All the opposition parties pointed out the defects in the design of the tax, the lack of comprehensiveness of the solution and its selectivity, and the need for a longer debate over the proposed policy.

The subject of taxation itself was debatable. There were proposals for alternative solutions, such as prohibiting the consumption of sugar [Confederation], taxing sugar itself [the Left] or other products rich in simple carbohydrates, such as sweet snacks or beer, and saturated fats, such as fast food [Confederation, KO]. It was also emphasized that although sweetened dairy drinks are sometimes rich in simple sugars, as are isotonic drinks and dietary supplements, they were excluded from the taxation [the Left]. It was argued that to realistically reduce the incidence of overweight and obesity by reducing the consumption of sweetened products, it would be necessary to expand the volume of products subject to the new levy [KO], to promote proper dietary choices [the Left], and to make these healthy choices more accessible by lowering their price, which may be done by reducing VAT rate [KO].

Assuming that an average citizen of Poland consumes 51 kg of sugar per year, of which 9 kg is consumed with sweetened beverages, the expected reduction in consumption with sweetened beverages could be around 20% (approximately 2 kg) [KO].

Another point of debate was the method of calculating the tax fee. Beverages imported directly by retail chains, which subsequently resell these beverages to consumers, were not supposed to be subject to the additional tax. This raised concern that it would result in bankruptcies among small retailers, apple, and raspberry juice producers, where Poland is one of the main suppliers [KO], and that it would have the adverse health effect, i.e., the absence of the surcharge would make sweetened carbonated beverages more affordable than natural juices [KO].

The purpose of the tax implementation was questioned by political opposition [KO, Confederation, the Left]. It was raised that, in reality, the tax does not arise from the care for the population's health but is rather an additional public levy [KO] and that it will be a tool to feed the low budget of the National Health Fund [KO]. It would be desirable for the pro-health interventions not to have a dispersed and sporadic character (such as the “healthy day at school”) but should be planned and implemented based on a system-level coordination pattern [KO]. It has been pointed out that part of the funds should be dedicated to the Ministry of National Education to organize more sports activities [KO].

Proceeding with the law raised doubts among opposition parties from the very beginning, including how public consultations were organized and how the Parliament finally passed the law. The government was accused of not registering the public consultations, and along with these consultations, further changes in the project were made, including the extension of the list of products excluded from taxation (e.g., beer). Except for the apparent nature of the consultation, this raises doubts about the purity of intentions [KO, Confederation]. The MPs also stressed that they received the bill only several hours before the Health Committee meeting intended to discuss the project [KO], and the entire legislative process was rushed [KO]. Furthermore, the opposition parties unanimously called for the extension of the vacatio legis, a constitutional principle of law [the Left] and will also reduce the negative impact on domestic manufacturers of sweetened beverages [the Left, the Confederation, the KO]. Finally, opposition parties claimed that the assumed implementation date (1 July 2020) is the middle of the fiscal year, which will cause unnecessary perturbations [Confederation].

## Discussion

Our research aimed to collect opinions expressed by stakeholders representing key areas connected with the implementation of a sugar tax and to identify the barriers and facilitators for its implementation in Poland. Between and within sectors, stakeholders expressed conflicting views on the effectiveness and potential socioeconomic consequences of implementing the SSB tax. However, all of them expressed views on the tax's advantages and disadvantages, identified potential barriers and opportunities, and presented suggestions for necessary changes that could enhance the positive effects of the implemented SSB tax.

First, the growing prevalence of overweight and obesity among the Polish population is an undeniable challenge for the national health policy, especially in the case of children and young people, who are the most susceptible to developing unhealthy eating habits. Hence, central level actions addressing this issue had been awaited by most stakeholders. However, they still found the SSB tax to be at most an additional funding stream for health promotion and overweight and obesity prevention activities, thus a complementary activity.

According to Statistics Poland, public expenditure on health care in Poland in 2020 amounted to 5.2% of GDP (30). The level of expenditure was somewhat lower according to Eurostat, amounting to 4.8% of GDP, which was the lowest level of expenditure among the European Union member states (31). Importantly, 2020 was an exceptional year because it was associated with the first wave of the COVID-19 pandemic, and as shown in Eurostat data, Poland was also the only country to reduce public spending on health care in 2020 (compared to 2019). As highlighted by most stakeholders, the SSB tax, implemented in 2021, is intended to serve as an additional funding source for healthcare activities. This is a kind of a major catalyst for this project, as public expenditure deficiencies have proven to be one of the key arguments in countries that have implemented similar food taxes in the past, e.g., France, Denmark, Finland, Hungary, Mexico, and several Pacific countries (32–35).

The study was conducted between July 2020 and May 2021. This was a unique moment because it was the time just before and shortly after implementing the SSB tax. Additionally, it was the first year of the COVID-19 pandemic, when the public debate concentrated highly on the health system performance, its underfunding and other disturbances, including the increase in overweight and obesity due to social isolation (36). Paradoxically, the sugar tax issue did not play a key role in these debates for two possible reasons.

First, in the first year of the pandemic, strong and restrictive measures were taken to contain the pandemic, including travel restrictions, limitations on social contact, and freezing of the economy. Naturally, this was the main focus for policymakers and the public (37).

Secondly, the most heated public debate regarding the sugar tax in Poland had already taken place before, along with the National Cancer Strategy announcing the implementation of such a tax in 2020 and originally assumed that the sugar tax would take into effect on 1 July 2020 (13). However, due to the delay in actual implementation, the public debate had been distracted. Additionally, no space for it was left due to the hasty procedure of enacting the law, which was subject to criticism by the political opposition.

There is a perception among health policy specialists that implementing the SSB is the easiest and most feasible health-related intervention, as it covers food products that are not considered the basis for a healthy and balanced daily diet; therefore, limiting their accessibility is desirable (27). The World Health Organization have expressed the same conviction (38). No case of questioning the legality of the SSB tax has been noted in Europe (32); however, in our study, Polish stakeholders raised numerous criticisms of this solution, addressing the subject of taxation (why sweetened beverages were chosen first), the burden of taxation (is the tax severe enough to discourage consumers), and the legislation procedure (lack of *vacatio legis* and general hastiness of legislation). Despite these objections, the majority of stakeholders who participated in our study expressed support for implementing the tax, except representatives of the industry and some economists. Interestingly none of the stakeholders referred to the issue of energy drinks taxation, which may be evidence for a wide acceptance of taxation of this category of products without specific reservations, or for a low priority for this category in the perception of stakeholders. The Ministry of Health expected discontent and declared that “*public information activities at the level of preliminary legislative work*.” However, as already mentioned, this debate was rather limited and unclear due to several circumstances. Additionally, it was not accompanied by any broader and comprehensive proposal regarding a healthy food environment that could convince the public of the rationality of the proposed solutions, as well as the willingness to stimulate reliable, substantive public discussion on the nutrition policy solutions with responsible citizen involvement, which is considered as one of the key determinants in creating real changes in consumer choices (39). Unfortunately, there was no such action in Poland when implementing the SSB tax, as well as at any time in the past, as evidenced by the regulation implemented in 2015 regarding collective nutrition of children in schools and kindergartens enforcing restrictive salt and sugar limits. The regulation resulted in a substantial lack of understanding among parents and a subsequent boycott leading to its eventual failure (40).

The sugar tax is considered a highly feasible solution. Existing studies show that this kind of fiscal solution in food policy is not dependent on the ideological background being implemented by both right-wing and left-wing parties (41). However, at the same time, this type of policy is highly dependent on political will, which is often lacking, especially in the face of a highly divided political scene and vital conflict (42). In Poland, since 2015, the governing majority has been relatively stable and had sufficient electoral support enough to pass any law proposed by the government. This was the case with the sugar tax. Undoubtedly this advantage increased the chances for the regulation to come into force. However, at the same time, it limits legislators' willingness to listen to arguments presented by representatives of the political environment and industry experts, and neither does it generate any need for wider public debate.

The producers' lobby was the most important opponent of the sugar tax and the loudest voice on its position. Its influence on the decision-making process resulting in an imbalance in public discourse was one of the main barriers to implementing the sugar tax identified in Australia and the United States (Richmond, El Monte and Telluride) (43, 44). Also, in Denmark, the industry has made significant contributions to the repeal of the “fat” tax (45, 46). On the other hand, a well-designed campaign promoting the SSB tax and bringing together stakeholders from both the industry and the scientific community may significantly improve the general acceptability of the project (33, 47).

Another aspect increasing the acceptability concerns the way sugar tax revenues are spent, especially if they are being spent to produce identifiable health effects. In Poland, the project assumed that the revenues would be distributed to the National Health Fund (NFZ) and local governments to carry out health education and promotion addressing overweight and obesity. Although this should be considered a desirable way of spending the collected funds, the prevailing view among stakeholders was that these measures might not increase the health system resources but, in reality, deplete the general government's budget.

After more than a year of implementing the sugar tax in Poland, it is still not possible to assess whether there have been any health effects. Some estimates suggest an impact on SSB purchases, as the tax generated revenues of PLN 1.5 billion (323 302 766,7 EUR) (see text footnote 1), which is much lower than the PLN 3 billion initially expected (48). There has been no tangible information about any concrete form of investing from the tax-generated resources in addressing overweight and obesity, which might be interpreted as evidence that the stakeholders' concerns were justified.

The sugar tax may be perceived as the subject of a multiple stream approach. This approach takes three separate perspectives: policy (the multitude of alternative solutions and selection of the one that is best), problem (the conditions that should be met) and politics (the ideology of political parties and public opinion) (49). If all three streams are consistent, a policy change window is created (50).

Our analysis of the Polish case reveals the following windows of opportunity:

- Problem stream: The SSB tax provides an additional stream of funding for the healthcare system, especially in terms of preventive interventions.

° An exceptional event is classified in this stream as a focusing event, i.e., an event that is sudden, possibly dramatic, including crises or disasters appearing in the social sphere and triggering meaningful impulses. This kind of event that caused confusion and dominated the attention of the public and decision-makers was the outbreak of the COVID-19 pandemic (51). Paradoxically the pandemic distracted the public, which made the tax debate difficult, but also made it easier to implement potentially unpopular solutions and reduced the risk of public discontent.

- Policy stream: a sound legal basis and expert and public support.
- Politics stream: political willingness and severity of the political conflict.

All of these windows are closely interrelated and interdependent. The quality of legislation depends on the political will, which is determined by the need for additional funds as well as the support of experts, public opinion and preferably—-other stakeholders. Being able to use all available windows of opportunity appears to be the key factor potentially creating space for the successful implementation of the policy and its continued effectiveness.

Our study has several important strengths, one of which is its time coincidence with actual implementation of the new levy in Poland. This connect the study with a specific project being proceeded, as well as with the real time social and political processes associated with the tax implementation. Another strength of our study is that it reflects a wide range of subjects, covering all the most important stakeholders, who may have any kind of interest connected with the SSB tax. Nonetheless, our study is not free from limitations, one of which is the indirect only reconstruction of the political parties' stances and opinions. Another limitation is resulting from the specificity of methodology applied, where there is no guarantee that opinions expressed by the interviewees are fully consistent with the views of all the representatives of given communities or organizations. Despite these limitations, our study constitutes a good foundation for further investigations, which primarily should consist of large-scale research addressing the issue of general public acceptance of the SSB tax, along with identifying of how the perception is changing over time. What should undoubtedly be investigated are also the actual health outcomes of the SSB tax, in the short term, as reflected by the changes in consumption, producers' and consumers' behavior and in the long term, to find whether a change in the health status of the population, especially obesity and dietary-related diseases prevalence, is affected by this kind of solutions.

## Conclusions

Based on the Polish experience related to the implementation of the sugar tax, and in particular, the perception of this project by the main stakeholders, several basic lessons can be learned. First, the most fundamental factor determining the political feasibility of the new law is to have a sufficient and stable parliamentary majority. However, successfully carrying out the legislative process is not a sufficient condition to ensure the actual success of the implemented policy if this success is seen as its socio-environmental acceptance, as well as achieving the assumed health-related aims of the SSB tax. To effectively achieve these kinds of objectives, it seems necessary to build a consolidated coalition with the community of health care experts, as well as to conduct an open and inclusive debate on the purposefulness of the implemented solutions and the use of the funds collected through the new tax. Opinions, as expressed by the stakeholders, seem to suggest that this process has been neglected in Poland.

Secondly, the vast majority of stakeholders generally agree with the need to apply fiscal solutions to limit the consumption of SSBs. As such, they constitute a group of potential allies in implementing the sugar tax. The exception in this respect are organizations representing the food industry, especially those specializing in the production of SSBs. However, the Polish experience shows that this requires effort on the side of public authorities to create and mobilize a strong coalition of support for the project and the active involvement of stakeholders in stimulating public debate. Ignoring them results in a perception that there is a lack of goodwill among political decision-makers and may also result in many difficulties at the stage of applying the new law, especially in light of the stakeholders' belief that the health aim of the new regulation is secondary to its fiscal aim. In addition to an effective information campaign at the project preparation stage, it also seems advisable to communicate the process of spending the resources obtained from the tax. The marginalization of stakeholders' participation in the project preparation also creates the risk of a defective tax structure, making the achievement of a real health-related aim even more questionable.

Finally, we found that a factor disrupting the process of harmonious preparation and implementation of the project is the high intensity of the political conflict, even if the main axis of the dispute is not related to the sugar tax. On the other hand, a favorable circumstance conducive to implementing the tax in Polish conditions was the emergence of other circumstances absorbing the public's attention to a greater extent. In Poland, such an element was the COVID-19 pandemics, with its consequences for the functioning of the health care system and its impact on the overall socio-economic life.

## Data availability statement

The raw data supporting the conclusions of this article will be made available by the authors, without undue reservation.

## Author contributions

KB, KK, and PR contributed to conception and design of the study. KB organized the database and wrote the first draft of the manuscript. KB, KK, OK, and PR performed the data analysis. All authors contributed to manuscript revision, read, and approved the submitted version.

## Funding

This research was conducted as a case study constituting part of the Work Package 6 of the Policy Evaluation Network (PEN) Project No. JFA PEN/I/PEN14/04/2019.

## Conflict of interest

The authors declare that the research was conducted in the absence of any commercial or financial relationships that could be construed as a potential conflict of interest.

## Publisher's note

All claims expressed in this article are solely those of the authors and do not necessarily represent those of their affiliated organizations, or those of the publisher, the editors and the reviewers. Any product that may be evaluated in this article, or claim that may be made by its manufacturer, is not guaranteed or endorsed by the publisher.

## Footnotes

^1^Calculated according to the exchange rate of the National Bank of Poland as of April 13, 2022.
