# Supplementary material for: Implementation of sugar-sweetened beverages tax and its perception among public health stakeholders. A study from Poland
Source: Front Nutr. 2022 Jul 29;9:957256. doi: 10.3389/fnut.2022.957256 (PMC9373029; doi:10.3389/fnut.2022.957256)
Supplement: Supplementary file 1 [file Table_1.DOCX]

Supplementary Material no 1- Initial set of codes:

1. **Stakeholders:**
2. Advantages
3. Disadvantages
4. Implementation:

- Circumstances favoring implementation
- Identified barriers
- Necessary conditions for introducing the tax

1. Respondent's opinion:
   - Suggestions for improvement of solutions
   - Alternative solutions
   - Experiences of other countries and lessons for Poland
2. Stakeholders:
   - Blocking stakeholders
   - Supporting stakeholders
   - Stakeholders that may have an impact
3. Expected results:
   - Impact on the occurrence of overweight / obesity
     1. Who is responsible for the occurrence of overweight / obesity?
   - Impact on the sale / consumption of beverages
   - Market and economic consequences
   - Use of obtained income / tax revenues /Opinia społeczeństwa
4. Anticipated public response:

- Means of relieving resistance
  - Tax as an interference with freedom of choice
  - Consumer groups particularly affected by the tax
  - Groups of consumers where the tax will be particularly helpful

1. Additional Notes

1. **Political parties:**
2. Procedure`s remarks
3. Effects :
   1. Budgetary impact
   2. Effects on consumption / obesity / overweight
   3. Impact on the market
4. Advantages
5. Disadvantages :
   1. Eliminating defects
